# Challenges Associated with Effective Implementation of CT Dose Check Standards and Radiation Monitoring Index in Computed Tomography: Healthcare Sector Experience

**DOI:** 10.3390/healthcare10101970

**Published:** 2022-10-08

**Authors:** Entesar Zawam Dalah, Yousuf Mohammad Al Musfari, Badriya Mohd Hassan Ali, Anwaar Abdulrahim Al Hammadi, Hashim Abdul Azeez Beevi, Manal Ibrahim Jaber, Alyazya Akeel Al-Ali, Ayesha Khalid Alkharoossi, Fairooz Hussain Al Hashemi, Fatma Mahmood Alkhatib, Sabaa Anam Aqil Khan

**Affiliations:** 1 HQ Diagnostic Imaging Department, Dubai Academic Health Corporation, Dubai P.O. Box 4545, United Arab Emirates; 2 College of Medicine, Mohammed Bin Rashid University, Dubai Academic Health Corporation, Dubai P.O. Box 505055, United Arab Emirates; 3 Department of Radiology, Rashid Hospital, Dubai Academic Health Corporation, Dubai P.O. Box 4545, United Arab Emirates; 4 Department of Radiology, Dubai Hospital, Dubai Academic Health Corporation, Dubai 7272, United Arab Emirates; 5 Department of Radiology, Hatta Hospital, Dubai Academic Health Corporation, Dubai P.O. Box 7272, United Arab Emirates; 6 Department of Radiology, Latifa Hospital, Dubai Academic Health Corporation, Dubai P.O. Box 9115, United Arab Emirates

**Keywords:** CT dose monitoring tools, CT Dose Check standard, radiation monitoring index, DOSE TQM, dose notification value, DRLs

## Abstract

Computed tomography (CT) radiation dose management tools should be used whenever possible, particularly with the increasing demand for acquiring CT studies. Herein, we aim to assess the advantages and challenges faced with implementing two CT dose management tools. A second aim was to highlight CT examinations exceeding dose notification values (NVs) and define the common set of causes. A total of 13,037 CT examinations collected over a six-month period, were evaluated, using two independent CT dose management tools, a CT Dose Notification prospective-view tool (PVT) following CT Dose Check standards and a retrospective statistical-based view tool (RSVT). Dose NVs were set to twice the Local Diagnostic Reference Levels. There was a significant discrepancy between dose NV counts registered with prospective (4.15%) and retrospective (7.98%) tools using T-Test. A core difference is the dose configuration setup, with PVT and RSVT being dose per series and whole study, respectively. Both prospective and retrospective dose management tools were equally useful despite their technical difference. Configuring the CT prospective dose notification check tool using NVs that is based on DRLs has limitations, and one needs to establish dose NVs per series to overcome this technical hurdle. Technical challenges make the implementation of CT Dose Check standards puzzling.

## 1. Introduction

Dose notifications and alert systems that trigger a dose notification pop-up or red flag when exceeding potentially high-radiation doses during radiological scans are very important, particularly for computed tomography (CT). Hence, radiation dose tracking and management tools should be implemented when available [1]. Such tools may exist in two forms: (a) a real time (prospective) monitor that is available on the CT system console itself known as the CT Dose Check standard [2], and (b) as independent automated software that can be used retrospectively to monitor dose metrics and scan acquisition parameters [3].

CT system scanners in compliance with the United States technical standard XR25 [2] offer a prospective “real-time” CT Dose Alert and CT Dose Notification [1,2,4]. The CT Dose Alert tool represents a vendor default sets of dose values that work as a radiation safety guard [2]. In contrast, the CT Dose Notification tool allows end-users to configure their CT machine to certain doses also known as predefined dose notification values [4]. Further, as per the American Association of Physics in Medicine (AAPM) medical physics practice guideline 6.a, radiation dose index monitoring systems recognized as software platforms can be used to retrospectively track and manage radiation dose indices together with image acquisition information and patient cumulative dose history [3]. Such platforms often offer statistical analysis of dose parameter values for a specific exam [1].

Notification values (NVs) also referred to as dose baselines or thresholds are established by healthcare providers based on their practice [5,6]. Configuring a CT system following CT Dose Checks standards (prospective tool) allow users to correct or confirm, in real-time, scan settings that might otherwise lead to unnecessarily high dose exposures to patients while undergoing CT examinations. CT Dose Checks standards is in place to prevent radiation accidents and achieve the desirable goal of eliminating radiation-induced risks [1,7].

Modern CT scanners provide and display dose product data in the form of: (a) computed tomography dose index (CTDI) and (b) dose length product (DLP). While the CTD dose index is being referred to and displayed in mGy, the second dose product is displayed in (mGy.cm) accounting for the scan length product in centimeters [8].

For the CT Dose Notification, a value for CTDI (in units of mGy) and DLP (in units of mGy.cm) can be used to trigger a notification when the scan yields a value that exceeds the predefined dose notification value (NV) for that prescribed scan [2,7]. Implementation of CT Dose Notification enables users to be more aware of the associated dose indices of the scan they are about to deliver [5]. When the system predicts that the predefined dose NV is to be exceeded for any of the prescribed scans, the user will be notified via a pop-up window prior to acquiring the actual scan and thus is required either to verify that the settings are correct or to change them. If the settings were correct, the operator could confirm the settings and proceed without additional action. In such a scenario, the operator can enter an explanatory comment before proceeding with the scan. The explanatory comment is often referred to as diagnosis reason or justification. While valuable, implementation of CT Dose Notification on a CT system console for real-time use is challenging [9]. More so if a clinic is to utilize both the prospective CT Dose Notification system and a retrospective dose monitoring tool to aid in patient dose optimization.

When utilizing CT dose management tools, either the one provided on the CT scanner console (i.e., prospective tool) or a statistical based dose management platform (i.e., retrospective tool), it is important to fully understand their basis, potentials as well as limitations. Herein, we aim to assess the potentials and limitations of the prospective and retrospective dose management tool as well as highlight similarities and discrepancies.

## 2. Materials and Methods

### Study Design and Data Collection

This retrospective study was approved by our Institutional Scientific Research Ethics Committee. CT studies and dose information were collected over 6 months period, from 1 August 2021 to 31 January 2022. A total of 3 healthcare institutes (denoted A, B, and C) and 6 different CT scanners (denoted A1, A2, A3, B1, B2, and C1) were enrolled in this study. Where A1, A2, and A3 are the CT scanners located at institute A, B1 and B2 are the CT scanners located at institute B and C1 is a single CT scanner located at institute C. A1 is a Siemens Somatom Force CT (Siemens Healthcare GmbH, Erlangen, Germany), A2 is a GE Medical Systems Discovery CT750 HD (General Electric Healthcare, Waukesha, WI, USA), A3 is a GE Medical Systems Optima CT660 (General Electric Healthcare Waukesha, WI, USA), B1 is a Siemens Somatom CT (Siemens Healthcare GmbH, Erlangen, Germany), B2 is a GE Medical Systems Revolution CT (General Electric Healthcare Waukesha, WI, USA), and C1 is a Siemens Somatom Definition AS + CT (Siemens Healthcare GmbH, Erlangen, Germany).

All 6 CT machines enrolled in this study were configured to a predefined dose NV, where NVs = 2 times our Local Diagnostic Reference Levels (DRLs). Hence, only CT exams with DRL values were considered, herein a total of 60 CT adult exams. Similarly, NVs were set to twice our Local DRLs on our retrospective statistical-based dose monitoring tool, DOSE TQM version 19.11 (Qaelum NV, Belgium) (Qaelum). CT studies exceeding the predefined dose NVs using the prospective view tool were automatically retrieved from each CT scanner on a weekly basis to prevent data loss. CT Dose Notification from CT system “prospective” will be referred to as CT dose logs. In contrast, CT studies exceeding the preset dose NVs using our retrospective statistical-based DOSE TQM were automatically retrieved on a monthly basis.

Justifications entered by CT radiographers’ when dose exceeded dose NVs on the CT system console were automatically exported to our statistical-based DOSE TQM dose management tool unless a technical error was encountered. CT studies exceeding the preset dose NVs using the DOSE TQM platform were resolved only by senior CT radiographers’ and further reviewed by a qualified medical physicist.

## 3. Results

### 3.1. Dose Configuration and Features

**Figure 1:**
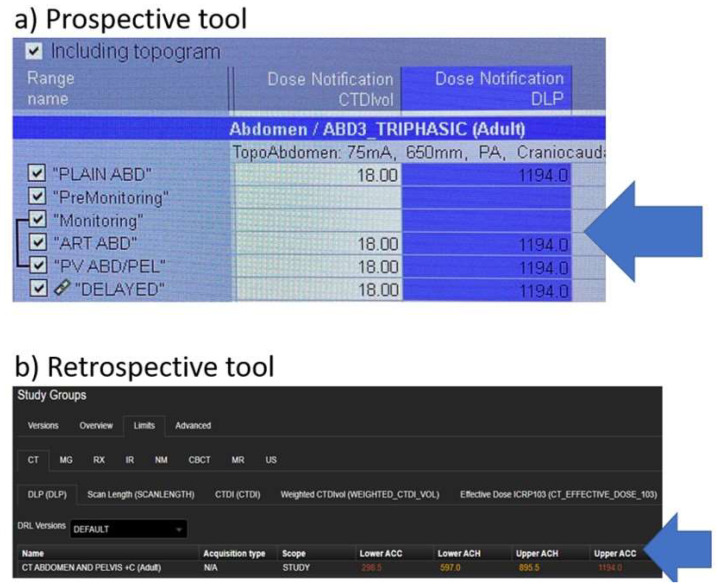
Demonstrates the key differences between the dose configuration setup for CT Abdomen/Pelvic + Contrast in a (**a**) prospective tool (CT system console) and (**b**) retrospective tool (DOSE TQM). a and b are screenshots taken straight from the CT system console and DOSE TQM, respectively.

A considerable difference was encountered while configuring “setting-up” the dose NVs on the prospective (CT system console) and the retrospective (statistical-based software, DOSE TQM) tools. While CT Dose Notification on the CT system console call for dose per series entry in order to trigger a dose notification value, the retrospective dose tool using DOSE TQM allows a dose limit entry that represents the whole study. Figure 1a,b demonstrates a technical fundamental difference between the prospective (CT system console) and retrospective (DOSE TQM) tools using CT Abdomen/Pelvis + Contrast exam as an example. In our healthcare sector, the predefined dose NVs for adult CT Abdomen/Pelvis + Contrast exam are set to CTDI = 18.0 mGy and DLP = 1194 mGy.cm. per our practice, CT Abdomen/Pelvis + Contrast can be acquired with two and up to 6 scan acquisition series [10]. Figure 1a demonstrates a CT exam performed with 6 scan acquisition series named PLAIN ABD (abdomen), PreMonitoring, Monitoring, ART ABD (arterial abdomen), PV ABD/PEL (porto-venous abdomen/pelvis), and DELAUED. NVs for CTDI and DLP were entered for 4 series except for the PreMonitoring and Monitoring. As shown in Figure 1a, the same CTDI and DLP NVs were entered for all of the 4 scan acquisition series. As mentioned in the methodology section, our predefined dose NV, where set at twice our Local DRLs, resulting in a single NVs for adult CT Abdomen/Pelvis + Contrast exam. In contrast, this is not an issue when configuring the retrospective tool. Retrospective tools allow dose configuration to the whole study or based on the protocol used, either way a single dose NV for each of CTDI and DLPs for a particular CT exam is sufficient. The 4 sets of dose NVs for adult CT Abdomen/Pelvis + Contrast shown in Figure 1b labeled as lower acceptable (ACC) set at 25th percentile of our Local DRL, Lower achievable (ACH) is our Local DRL, upper ACH is set at the 75th percentile of our Local DRL, and the upper ACC is set at twice our Local DRL.

Not only was the dose configuration setup different in our prospective (CT system console) and retrospective (statistical-based software, DOSE TQM) tools, but the features and functions provided on both dose management tools were also different. Table 1 summarizes feature differences between the two CT dose management tools, CT system console (prospective) and DOSE TQM (retrospective).

### 3.2. Dose Notification Counts

**Figure 2:**
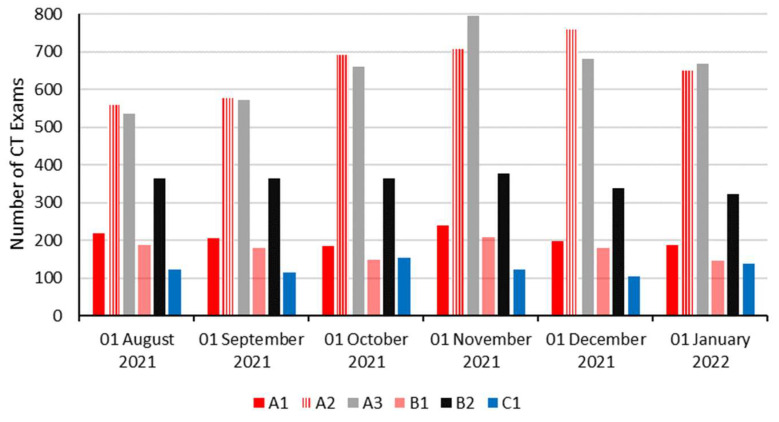
Demonstrates the total number of studies per institute per scanner for patients undergone CT studies across the 6 CT scanners during the 6-month period.

**Figure 3:**
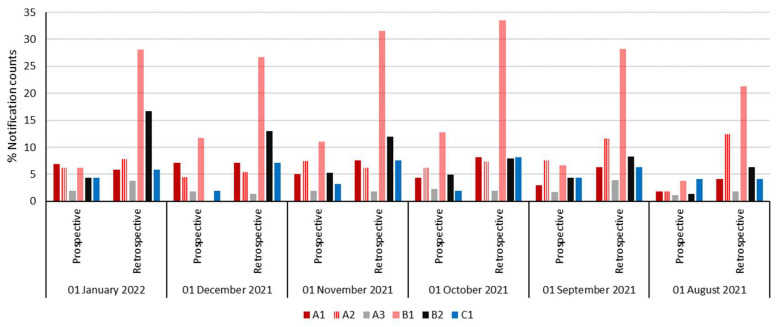
Demonstrates the percentage of notification counts using both tools prospective, i.e., CT console view tool and retrospective, i.e., DOSE TQM view tool for patients undergone CT studies across the 6 CT scanners during the 6-month period.

During the 6-month period, a total of 13,037 CT studies (Figure 2) were carried out on all 6 CT scanners (A1, A2, A3, B1, B2, and C1). The number of notification counts registered through the retrospective dose minoring (DOSE TQM) tool were exceeding the number of notification counts registered through the prospective dose monitor (CT system console). With the percentage of cases that exceeded predefined dose NVs, including CTDI and DLP being 4.15% (CT system console, prospective, tool) and 7.98% (DOSE TQM, retrospective, tool), see Figure 3. The percentage notification counts per scanner that were registered on the CT system console (prospective) is demonstrated in Figure 3. The % notification counts were calculated as (total CT logs divided by the total number of CT examinations acquired). CT exams with no DRL values were excluded. Comparatively, Figure 3 details the percentage of notification occurrences per scanner using the DOSE TQM (retrospective) during the 6-month period. Heterogenous % notification count distribution was noticed across the different CT scanners during the 6-month period for both dose alert tools (Figure 3).

**Figure 4:**
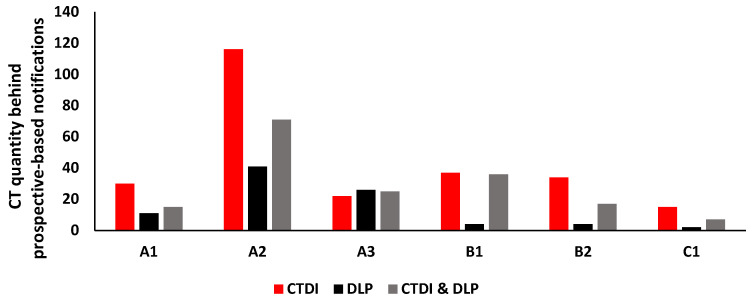
Demonstrates the CT dose quantities associated with notification counts using the prospective tool (CT system console) across the 6 CT scanners during the 6-month period.

Analyzing dose notification counts revealed that the majority of dose notifications triggered were attributed to CTDI dose quantity, particularly on the CT system console prospective dose management tool, see Figure 4.

### 3.3. Notification Studies

CT studies with notable dose notification counts are demonstrated in Table 2 for both the CT system console (prospective) and DOSE TQM (retrospective) dose management tools throughout the 6-month period. For the CT system console, CT Abdomen/Pelvis + Contrast exam was noted to have the most dose notification counts followed by CT Chest/Abdomen/Pelvis + Contrast, CT cardiac angiogram, CT kidney ureter bladder (KUB), and CT chest exams. In contrast, using the DOSE TQM, the highest registered dose notification counts were for CT polytrauma and CT brain with computed tomography angiogram (CTA) followed by CT Abdomen/Pelvis + Contrast, CT Chest/Abdomen/Pelvis + Contrast, CT cardiac angiogram, CT KUB, and CT chest exams.

### 3.4. Radiographer Justifications

The most common diagnosis reasons and justifications used by our CT radiographers’ using both dose management tools are summarized in Table 3. Besides patient size, patient mispositioning “off-center” and blind scans “out of scout” were commonly seen justifications on the retrospective (DOSE TQM) dose management tool. While patient size is a mutual finding on both the prospective and retrospective dose management tools, high scan length due to added delay scan or to cover pathology or because of a combined study as well as arms in the field of view were frequently reported in the prospective dose management tool together with technical errors. The justifications reported by our CT radiographers’ using the retrospective (DOET TQM) dose management tool seem to be matching to a certain degree the justification reported by [9] using their retrospective DoseWatch platform.

## 4. Discussion

Achieving adequate diagnostic image quality that addresses clinical concerns in the most dose efficient manner is a goal every radiology department is diligent to achieve. It is also a requirement that is recommended by International Commission of Radiation Protection [11], the International Atomic Energy Agency [12], and the World Health Organization [13]. With this, utilizing and implementing dose tracking/management tools is desirable whenever available. Such tools offer an opportunity to adjust and pinpoint areas in need of improvement within a clinical practice. Herein, patient mispositioning “off-center” counted for 30% of the total justification used by our CT radiographers. A similar finding was reported by [9]. It is well known that patient mispositioning compromises CT tube current modulation function [14], leading indirectly to increased patient exposure [15]. Since a majority of commercial CT scanners are not equipped with 3D cameras to assist CT radiographers in patient positioning, patient centering remains manually and subjected to an individual’s level of experience and skills. Manually adjusting vertical and horizontal offset is particularly challenging for large sized patients as well as for extremely thin patients. Moreover, the positioning evaluation tool provided in the retrospective dose management software does not apply to certain body parts such as extremities and the brain. Such challenges are being recognized by international guidelines that emphasize the need for continuous staff training remains vital [1].

In an effort to attain patient dose optimizations across our healthcare facilities, dose NVs were predefined based on our Local DRL values, implemented, and utilized using two independent dose management tools, a prospective (CT system console) and retrospective (DOSE TQM). The CT Dose Notification prospective tool allows for real-time individual dose monitoring that is not limited to certain audit cycles [9].

The present paper reports a number of interesting findings, first the % notification alert counts associated with our prospective tool were significantly lower than the % notification alerts associated with our retrospective tool, a factor of 2. Such a finding is attributed to the fundamental difference in setting-up “configuring” dose NVs on both tools, this was clearly demonstrated in Figure 1. The dose configuration setup in the prospective tool (CT system console) calls for dose value alert entry per series (Figure 1a) unlike the dose configuration setup in the retrospective tool (DOSE TQM). The latter calls for a single dose notification value entry that represents the whole study (Figure 1b). Clearly, such a discrepancy in dose notification value will impact the % notification alerts. To further clarify this let us consider a CT exam that could be acquired with more than one series “image acquisition”. A good example is CT Abdomen/Pelvis + Contrast exam. The single NV used to trigger dose notification was predefined in our institute to CTDI: 18 mGy and DLP: 1194 mGy.cm. In cases where medical concerns call for multi-scan series, the NV on the CT system console was fixed to CTDI: 18 mGy and DLP: 1194 mGy.cm for every scan series (Figure 1a). DLP of every abdomen series may range between 400 to 500 mGy.cm depending on patient size, pathology, contrast, and acquisition scan parameters [10]. In this case, there will be no DLP dose trigger on the CT system console as every series is below the predefined NV 1194 mGy.cm. With such a setup, the overall % notification counts will certainly be less using the prospective (CT system console) tool. In addition, one should expect most of the dose notification counts triggered on the CT system console to be CTDI related. Figure 4 confirms our expectation with CTDI being the common dose notification trigger across all 6 CT scanners during the 6-month period. One should not forget that patient size also generates CTDI notifications. Along these lines, CT polytrauma and CT brain with CTA were not listed among the CT studies causing dose notifications on the prospective (CT system console) tool. Our NVs for polytrauma (CTDI: 34 mGy and DLP: 3936 mGy.cm) and brain with CTA are (CTDI: 49.6 mGy and DLP: 4900 mGy.cm). In consideration of this, CT system console dose prospective tool may be helpful in real-time monitoring and assessing compliance with dose NV levels [16]. However, it may result in rather an underestimation of dose notification alert counts if CT dose notifications were configured similar to the present study. Hereafter, high-end CT dose studies may go undetected.

In light of this, can one consider multi-scan series or the number of scan series as a valid justification when commenting on a CT system console prospective triggered notification alert? Since prospective tools call for dose NV entry per series, the number of series, in this case, is not behind the triggered notification. In this case, dose per scan series is affected by other parameters such as patient size, scan length, scan acquisition parameters, positioning, pathology, patient habits, and contrast media. This is particularly true for CT studies covering multi anatomical locations such as CT Chest/Abdomen/Pelvis. In such scenarios, should one base dose NVs on a body part or the whole study, i.e., DRLs? Another recognized challenge is the limited references providing dose benchmarks based on CT protocols let alone series.

A second finding is the discrepancy in the type of CT studies that registers high notification counts using both tools. Our statistical-based retrospective dose management tool allows comprehensive comparison between patients scanned on the same device and undergone the same CT study. Moreover, our retrospective tool is facilitated with positioning and blind scan evaluation tools. All of which facilitate accurate diagnosis and justification of why certain cases exceeded a predefined dose NV. On the other hand, diagnosis and justifications using the prospective dose management tool depend highly on CT radiographer’s level of expertise and understanding of scientific fundamentals as well as the technology [17]. CT Dose Notification prospective tool is not designed to assist inclusive comparison, nor that positioning evaluation tool is provided except for some advanced CT scanners that are facilitated with 3D cameras.

Dose monitoring and management tools have the potential to revolutionize quality assurance in diagnostic imaging and can open pathways to various research opportunities. Therefore, when aiming for high standards in quality and patient care, implementing dose monitoring tools can become a dire necessity. With nearly half of the American College of Radiology dose index registry participant demonstrating a lack of familiarity with CT Dose Check standards [18], raises an urgent need for international discussion and action on CT Dose Notification “dose per series” benchmark values and means of clinical implementation [19]. Recently, the National Electrical Manufacturers Association (NEMA) published a thorough Dose Check standard covering all relevant aspects of CT [20]. At present, there is no specified CT Dose Notification standard [21]. Food and Drug Administration proposed a CT Dose Alert value for CTDI of 1000 mGy [21]. Technical challenges and knowledge gaps encounter the feasibility of effectively implementing dose monitoring and management tools in a clinical practice. Such observation was supported by [9,22].

To date, a limited number of studies addressed the challenges and limitations associated with the commonly increased requirement of implementing dose monitoring tools in radiological practices. Furthermore, only a few studies looked at the potential of using dose alert tools such as DoseWatch in investigating instances of increased radiation doses [9,23]. Howard and colleagues [4], investigated the impact of using CT Dose Check standard on the daily workflow.

To our knowledge, we are the first to comply with CT Dose Check standards in our nation. All of our CT scanners were configured to a CT Dose Notification system to effectively monitor patient dose in real-time. Further, we utilize a statistically inclusive retrospective dose management platform across all our healthcare facilities that incorporate CT scanners.

Several limitations were encountered in the present study, first and foremost the data represented here does not represent our entire CT clinical services. Only adult CT studies with established DRL values were included. Further, CT Dose Notification of the CT Dose Check standards was configured using dose NVs based on the whole study despite the fact that it should be series based. Particularly, since CT practice encompasses several examination types other than the plain CT routine body and head examinations. To this end, several initiatives have begun in order to address these challenges in our practice. Ongoing progress to establish dose baseline per series for the common protocols used to acquire CT studies is listed in Table 2.

## 5. Conclusions

CT dose monitoring tools, including the prospective CT Dose Check standard and retrospective radiation monitoring index, have proven to be an important part of an institute’s CT operation. In addition to offering high radiation safety, such tools can help maintain image quality at an acceptable radiation dose level. Configuring the prospective CT Dose Check standard to a dose NVs that is based on DRLs is with limitations; thus, one needs to establish dose NVs per series to overcome this technical hurdle. Owing to the continuous improvement and technical developments, implementation of newly operational dose monitoring tools requires a justification system that is continuously evolving and updated.

## Figures and Tables

**Table 1:** Summary of feature differences between the CT console (prospective) and DOSE TQM (retrospective) dose monitoring tools.

Feature	CT Console (Prospective)	DOSE TQM (Retrospective)
NV can be entered for both CT dose quantities, CTDI and DLP	Yes	Yes
Dose NV configured per series/phase	Per series/phase	Whole study
Different NV values can be used depending on the series or phase	Yes	Yes (e.g., upper ACC/ACH and lower ACC/ACH) but for the whole study
Statistical comparison to aid	No	Yes
Patients’ accumulative dose history	No	Yes
Allows dose alteration/reduction	Yes	No, it’s retrospective
Positioning evaluation tool	No, limited scanners	Yes, very effective
Credentials needed to override NV alerts	No	No
Comparison using the same device, same study description, and even same protocol	No	Yes

^ACC^ Refer to acceptable dose, ^ACH^ Refer to achievable dose.

**Table 2:** Common CT studies exceeding NVs using both the CT system console (prospective) and DOSE TQM (retrospective) dose monitoring tools.

Common CT Exam	
CT Console (Prospective)	DOSE TQM (Retrospective)
CT Abdomen/Pelvis + Contrast	CT Polytrauma
CT Chest/Abdomen/Pelvis + Contrast	CT Brain Stroke With CTA
CT Cardiac Angiogram	CT Abdomen/Pelvis + Contrast
CT KUB (Kidney Ureter Bladder)	CT Chest/Abdomen/Pelvis + Contrast
CT Chest/CT Chest + Contrast	CT Cardiac Angiogram
CT Chest Pulmonary Angiogram	CT KUB (Kidney Ureter Bladder)
	CT Chest/CT Chest + Contrast
	CT High Resolution

^CTA^ Refer to computed tomography angiogram.

**Table 3:** Common Justifications used by our CT Radiographers’ using the prospective CT Dose Notification and retrospective DOSE TQM monitoring tools.

Our Institutional Standardized Justifications				Radiographers’ Common Justifications	
CT System Console (Prospective)		DOSE TQM (Retrospective)			
Actual or Suspected Justification	Justification	Notification Type	Justification	CT System Console (Prospective)	DOSE TQM (Retrospective)
Urgent case “No time to write a justification note”	In the diagnosis reason window type 1	CTDI	Off center > 15 mm; Patient size-obese; Pitch < 1 Hands down; High kV due to (beam hardening, non-typical scan, patient size, pitch, off center, hands down); Combined study	Patient size-obese	Patient size-obese
Patient size-obese	Obese patient	DLP	High CTDI; High scan length	High scan length—added delay scan(s)	Off-center
Off center	To include ROI (e.g., Knee/Ankle/Foot); External fixator; Difficult positioning (e.g., bulky patient, big abdomen, restless patient, trauma patient, uncooperative patient, etc.); Female patient positioned by male radiographer			High scan length—cover pathology	Blind scan (out of scout)
High scan length	Due to patient height; To cover pathology; Radiologists request; Add delay scan (s); Repeated scan due to artifact (e.g., breathing, movement), technical error, etc. Combined study			High scan length—combined study	
Alternative positioning	Disoriented patient; Arms in the FOV			Alternative positioning—arms in the FOV	
Non-typical scan	Due to using: Dual energy; 4D-Care Dose Modulation; Pitch adjusted; Combined scan			Beam hardening—metallic implant	
Beam hardening	In case of: Metallic implant; Metallic ornament; External fixator Metallic item in FOV			Repeated—technical error	

^ROI^ Refer to region of interest; ^FOV^ Refer to field of view; ^ACC^ Refer to acceptable dose; ^ACH^ Refer to achievable dose.

## Data Availability

Not applicable.

## References

[1] Cody D., Dillon C., Fisher T., Liu X., McNitt-Gray M., Patel V. (2021). AAPM Medical Physics Practice Guideline 1.b: CT protocol management and review practice guideline. J. Appl. Clin. Med. Phys..

[2] American Association of Physicists in Medicine (2011). AAPM Recommendations Regarding Notification and Alert Values for CT Scanners: Guidelines for Use of the NEMA XR 25 CT Dose-Check Standard. http://www.aapm.org/pubs/CTProtocols/documents/NotificationLevelsStatement_2011-04-27.pdf.

[3] Gress D.A., Dickinson R.L., Erwin W.D., Jordan D.W., Kobistek R.J., Stevens D.M., Supanich M.P., Wang J., Fairobent L.A. (2017). AAPM medical physics practice guideline 6.a.: Performance characteristics of radiation dose index monitoring systems. J. Appl. Clin. Med. Phys..

[4] Howard M., McCollough C., Leng S., Yu L., Bruesewitz M. (2014). Use of CT Dose Notification and Alert Values in Routine Clinical Practice. J. Am. Coll. Radiol..

[5] Brill P., Hentel K., Mahmood U., Min J., Min R., Phillips C. (2012). GE Blueprint A Guide to CT Radiation Dose Management Developed in conjunction with Weill Cornell Imaging at New York–Presbyterian Lower Dose by Design. Weill Cornell Imaging at NewYork-Presbyterian. https://services.gehealthcare.sa/.

[6] Denison K. (2011). Dose Check Overview. GE Healthcare. http://www.gehealthcare.com/LowerDoseByDesign.

[7] Vano E., Fernández J., Ten J., Sanchez R. (2022). Benefits and limitations for the use of radiation dose management systems in medical imaging. Practical experience in a university hospital. Br. J. Radiol..

[8] Boone J.M., Strauss K.J., Cody D.D., McCollough C.H., McNitt-Gray M.F., Toth T.L. (2011). Size-specific dose estimates (SSDE) in pediatric and adult body CT examinations. American Association of Physics in Medicine (AAPM TG-204).

[9] Crowley C., Ekpo E.U., Carey B.W., Joyce S., Kennedy C., Grey T., Duffy B., Kavanagh R., James K., Moloney F. (2021). Radiation dose tracking in computed tomography: Red alerts and feedback. Implementing a radiation dose alert system in CT. Radiography.

[10] Dalah E.Z., Alsuwaidi J.S., Hamed M.S., Gani A.H.A., Beevi H.A.A., Panangatil A.G., Funtelar C.O., Ferrer A.Y., Al Hussein S.G.A.B., Albedwawi S.A. (2022). Challenges Experienced in Establishing Clinical Indication Based Diagnostic Reference Levels: Pilot Study. Eur. J. Radiol..

[11] Vañó E., Miller D.L., Martin C.J., Rehani M.M., Kang K., Rosenstein M., Ortiz-López P., Mattsson S., Padovani R., Rogers A. (2017). ICRP Publication 135: Diagnostic Reference Levels in Medical Imaging. Ann. ICRP.

[12] IAEA: SSG-46 (2018). Radiation Protection and Safety in Medical Uses of Ionizing Radiation.

[13] Lau L., Perez M. (2008). Global Initiative on Radiation Safety in Healthcare Settings. Technical Meeting Report. 15th to 17th December.

[14] Marsha R., Silosky M. (2017). The effects of patient positioning when interpreting CT dose metrics: A phantom study. Med. Phys..

[15] Barreto I., Lamoureux R., Olguin C., Quails N., Correa N., Rill L., Arreola M. (2019). Impact of patient centering in CT on organ dose and the effect of using a positioning compensation system: Evidence from OSLD measurements in postmortem subjects. J. Appl. Clin. Med. Phys..

[16] Parakh A., Euler A., Szucs-Farkas Z., Schindera S. (2017). Transatlantic Comparison of CT Radiation Doses in the Era of Radiation Dose–Tracking Software. Am. J. Roentgenol..

[17] Maldjian P., Goldman A. (2013). Reducing Radiation Dose in Body CT: A Primer on Dose Metrics and Key CT Technical Parameters. Am. J. Roentgenol..

[18] Miller D., Bhargavan-Chatfield M., Armstrong M., Butler P. (2014). Clinical Implementation of the National Electrical Manufacturers Association CT Dose Check Standard at ACR Dose Index Registry Sites. J. Am. Coll. Radiol..

[19] Loose R.W., Vano E., Mildenberger P., Tsapaki V., Caramella D., Sjöberg J., Paulo G., Torresin A., Schindera S., Frija G. (2020). Radiation dose management systems—requirements and recommendations for users from the ESR EuroSafe Imaging initiative. Eur. Radiol..

[20] NEMA (2019). Computed Tomography Dose Check. https://www.nema.org/Standards/view/Computed-Tomography-Dose-Check.

[21] Mahesh M. (2016). What Is the CT Dose Check Standard, and Why Do CT Scanners Need to Be in Compliance?. J. Am. Coll. Radiol..

[22] Szczykutowicz T., Bour R., Ranallo F., Pozniak M. (2018). The Current State of CT Dose Management Across Radiology: Well Intentioned but Not Universally Well Executed. Am. J. Roentgenol..

[23] Osman N.D., Isa S.M., Karim N.K., Ismail N., Roslee M.A., Naharuddin H.M., Razali M.A. (2020). Radiation dose management in CT imaging: Initial experience with commercial dose watch software. J. Phys. Conf. Ser..

